# Adenocarcinoma of Lung Presenting as Lambert-Eaton Myasthenic Syndrome

**DOI:** 10.1177/2324709617721251

**Published:** 2017-07-14

**Authors:** Sumera Bukhari, Rabia Soomro, Shaikh Fawwad, Chikezie Alvarez, Sara Wallach

**Affiliations:** 1 St. Francis Medical Center, Seton Hall University, Trenton, NJ, USA

**Keywords:** Lambert-Eaton myasthenic syndrome, paraneoplastic syndrome, adenocarcinoma of lung, neuromuscular disorder, lung cancer

## Abstract

Lambert-Eaton myasthenic syndrome (LEMS) is a paraneoplastic neuromuscular junction disorder. LEMS presents with muscular weakness and fatigability, mainly involving the proximal lower limbs. There are 2 types of LEMS depending on the etiology: paraneoplastic and idiopathic. The paraneoplastic form, which constitutes more than a half of the cases, is mostly associated with intrathoracic neoplasms. Most cases are seen in patients with small cell lung cancer; other subtypes of lung cancer are extremely rare. In this article, we report a case of LEMS as a rare association with adenocarcinoma of the lung.

## Introduction

Lambert-Eaton myasthenic syndrome (LEMS) is a rare paraneoplastic disorder of neuromuscular junction (NMJ), characterized by muscle weakness and fatigability, mainly involving the proximal lower limbs. LEMS is mostly associated with small cell lung cancer and is rarely associated with other types of lung cancers. A PubMed and Google Scholar literature search discovered only 5 published cases (Table 1) in the literature (English), reporting LEMS associated with pulmonary adenocarcinoma.^1-5^ In this article, we report a rare case of LEMS associated with adenocarcinoma of the lung.

**Table 1.** Case Reports: Lambert-Eaton Myasthenic Syndrome Associated With Lung Adenocarcinoma.

Case No.	First Author	Year	Age	Sex	Site	Histological grade	Treatment	Outcome
1	Ramos-Yeo^1^	1987	56	Male	RLL	Poor	Steroid, plasmapheresis	Death at 2 years due to infection
2	Sumitomo^2^	1989	58	Male	RML	Poor	Lobectomy, adjuvant chemotherapy	Surviving at 2 years of diagnosis
3^a^	Okudera^3^	1996	32	Male	RUL	Poor	Chemotherapy	Death at 5 months due to DIC
4^a^	Milanez^4^	2008	66	Male	RUL	Moderate	Lobectomy	Death at 16 days due to sepsis
5	Arai^5^	2012	75	Male	RLL	Moderate	Lobectomy	Surviving at 4 months
6	This case report	2017	78	Female	RUL	Moderate	Palliative care	Death within 2 weeks of diagnosis

Abbreviations: RLL, right lower lobe; RML, right middle lobe; RUL, right upper lobe; DIC, disseminated intravascular coagulation.

a In Japanese literature with English abstract.

## Case Presentation

A 78-year-old Caucasian female with a past medical history of hypertension presented to the emergency department with complaints of lower extremity weakness for last 2 to 3 months, which has been progressively worsened. The patient denied any numbness, paresthesias, memory loss, trauma, dizziness, tremors, history of seizures, family history of neurological disease, or loss of consciousness. The patient also denied having any bowel or urinary incontinence. She also experienced a 5-pound unintentional weight loss over a 2-month period. She denied smoking, drinking alcohol, or abusing any drugs. Her medications were aspirin 81 mg oral daily and amlodipine 5 mg oral daily. She denied use of any illicit drug or herbal supplement.

On physical examination, vital signs were the following: blood pressure of 139/79 mm Hg, pulse rate of 85 beats per minutes, respiratory rate of 17 breaths per minute, temperature of 98.6°F, and an oxygen saturation of 99% on room air. She was alert and oriented to person, place, and time. Pertinent positive findings on neurological examination showed motor weakness of her lower extremities muscle with power of 3 out of 5 in her proximal lower extremities’ muscles. Her results for manual muscle strength testing increased from 3 out of 5 to 4− out of 5 for her bilateral lower extremities after a sustained 30-second contraction. The sensations were intact to light touch and pinprick. Deep tendon reflexes were 2+ bilaterally at the patella, ankle and 1+ bilaterally at the biceps and triceps, and Babinski’s sign was down-going bilaterally. There were no cerebellar signs. Cranial nerve examination was intact II-XII. There was no involvement of extraocular muscles or respiratory compromise. The remainder of the systemic physical exam was unremarkable.

**Figure-1.**
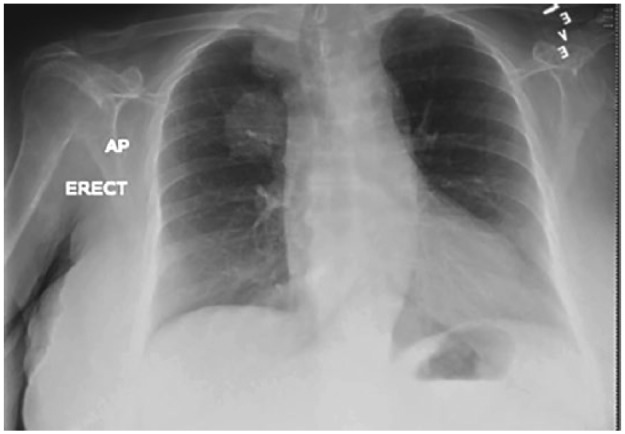
Chest X-ray showing a 4.3 cm right upper lobe lung mass.

**Figure-2.**
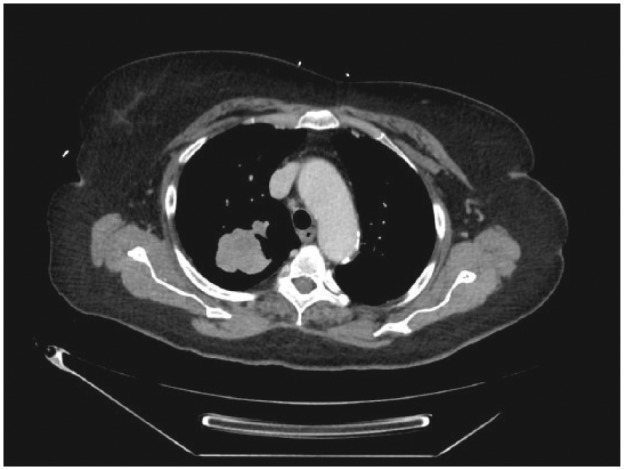
Computed tomography scan of the chest showing a right upper lobe lung mass.

Laboratory work showed no metabolic abnormality or signs of anemia. Renal and liver function test were within normal range. Routine chest X-ray showed a 4.3 cm right upper lobe lung mass posteriorly in the supra-hilar region and a 0.8 cm right upper lobe lung nodule (Figure 1). Subsequent computed tomography (CT) scan of the chest confirmed the lung mass with a maximal diameter of 4.9 cm, most likely a primary lung malignancy with a smaller satellite nodule within the medial apical aspect of the right upper lobe (Figure 2). A CT scan of the brain showed enhancing lesions within the occipital and temporal regions of brain consistent with metastatic disease. The patient, however, did not have any upper motor neuron signs on neurological exam. Patient’s clinical symptoms were consistent with lower motor neuron involvement at the NMJ, being purely motor with no sensory deficits. CT-guided biopsy of the lung mass revealed invasive adenocarcinoma moderately differentiated. LEMS was the most likely explanation for her proximal lower limb weakness as a paraneoplastic syndrome secondary to adenocarcinoma of the lung. Furthermore, the electromyogram and antibodies test to voltage gated calcium channel were ordered to confirm LEMS. However, understanding the extent of the disease with poor prognosis and patient’s functional status with deteriorating condition, the outcome was discussed with family by the oncology team and the family decided not to proceed with chemotherapy, and opted for palliative care. The patient underwent few session of palliative radiation therapy. Subsequently, the patient was referred to inpatient hospice where the patient died within few days.

## Discussion

Lambert-Eaton myasthenic syndrome was first described as a paraneoplastic syndrome in patients with lung cancer, now known to be idiopathic in nearly half the cases.^6^ Paraneoplastic LEMS is usually observed in older patients, with smoking history and who have developed small cell lung cancer.^7^ However, our patient was a nonsmoker and had adenocarcinoma of the lung. Idiopathic LEMS tends to occur in younger patients and nonsmokers.^8^ History of smoking and age at onset may predict the coexistence of neoplasm once LEMS is diagnosed and should prompt an aggressive search for underlying cancer. The actual incidence of LEMS may be higher than what is reported in the literature as symptoms may erroneously be attributed to extreme weight loss, peripheral nerve and central brain involvement, or the effects of treatment.

LEMS should be distinguished from myasthenia gravis (MG) to avoid misdiagnosis, as both have similar presentation but can be distinguished by relative sparing of ocular and bulbar muscles, which are more prominently involved in MG. The primary symptoms in LEMS are lower extremity weakness, generalized fatigue, muscles ache, and sometimes tenderness.^6^ In our patient, there was no involvement of ocular or bulbar muscles, and the main symptom was bilateral proximal lower extremities muscular weakness, which improved with sustained contraction. LEMS can also manifest autonomic dysfunction like dry mouth, postural hypotension, impotence, blurred vision, and constipation in up to 75% of patients.^9^

LEMS may present 2 years earlier than the diagnosis of associated malignancy. Therefore, it can contribute to the identification of occult cancer. Other conditions associated with LEMS include hypothyroidism, pernicious anemia, celiac disease, and juvenile-onset diabetes mellitus.^10^ Blood work is often useful in clarifying the diagnosis and checking creatinine kinase; thyroids functions may aid in narrowing down differentials. A chest CT should be part of evaluation because of association with lung cancer.^10^ The presence of antibodies to presynaptic P/Q-type and N-type voltage gated calcium channel at the NMJ and incremental pattern of electromyogram can be helpful in the diagnosis of LEMS. However, these antibodies may be found in patients with systemic lupus erythematosus, rheumatoid arthritis, or MG.^11,12^ LEMS has also been associated with other araneoplastic autoantibodies including CRMP-5; anti-glutamic acid decarboxylase antibodies; anti-neuronal nuclear antibody, type 1 (Hu); anti-neuronal nuclear antibody, type 2 (Ri); anti-glial nuclear antibody, type 1 (AGNA1, Sox1); amphiphysin antibody; and Zic4.^13-18^

Treatment of LEMS must be custom-made per disease severity, comorbidities, coexistence of cancer, and life expectancy. Since LEMS associated with cancer is a paraneoplastic phenomenon, primary treatment should be targeted to cancer because LEMS frequently improves with successful cancer therapy. Immunotherapy of LEMS itself usually produces little or no improvement in strength without effective treatment of underlying malignancy.^19,20^ Pyridostigmine has been used for LEMS with some success, and the use of plasma exchange has had inadequate success as the response is short-lived in LEMS as compared to other diseases such as MG.^21^

## Conclusion

LEMS is a rare autoimmune but the most common neurological paraneoplastic syndrome. The identification and aggressive treatment of an inducing malignancy are the priority. Clinically, patients with muscle weakness must be managed by physicians while considering LEMS as a differential diagnosis. If no tumor is found, patients should repeatedly be evaluated for occult malignancy based on the risk factors for cancer. Therefore, screening for malignancy is strongly recommended for suspected LEMS.
